# Regional pulmonary effects of bronchoalveolar lavage procedure determined by electrical impedance tomography

**DOI:** 10.1186/s40635-019-0225-6

**Published:** 2019-02-15

**Authors:** Inéz Frerichs, Peter A. Dargaville, Peter C. Rimensberger

**Affiliations:** 1 Department of Anaesthesiology and Intensive Care Medicine, University Medical Centre Schleswig-Holstein, Campus Kiel, Arnold-Heller-Str. 3, 24105 Kiel, Germany; 2 Neonatal and Paediatric Intensive Care Unit, Royal Hobart Hospital, Hobart, Australia; 3 Menzies Institute for Medical Research, University of Tasmania, Hobart, Australia; 4 Pediatric and Neonatal Intensive Care Unit, Children’s Hospital, University of Geneva, Geneva, Switzerland

**Keywords:** EIT, Electrical bioimpedance, Functional imaging, Ventilation monitoring, Regional ventilation, Alveolar collapse, BAL

## Abstract

**Background:**

The provision of guidance in ventilator therapy by continuous monitoring of regional lung ventilation, aeration and respiratory system mechanics is the main clinical benefit of electrical impedance tomography (EIT). A new application was recently described in critically ill patients undergoing diagnostic bronchoalveolar lavage (BAL) with the intention of using EIT to identify the region where sampling was performed. Increased electrical bioimpedance was reported after fluid instillation. To verify the accuracy of these findings, contradicting the current EIT knowledge, we have systematically analysed chest EIT data acquired under controlled experimental conditions in animals undergoing a large number of BAL procedures.

**Methods:**

One hundred thirteen BAL procedures were performed in 13 newborn piglets positioned both supine and prone. EIT data was obtained at 13 images before, during and after each BAL. The data was analysed at three time points: (1) after disconnection from the ventilator before the fluid instillation and by the ends of fluid (2) instillation and (3) recovery by suction and compared with the baseline measurements before the procedure. Functional EIT images were generated, and changes in pixel electrical bioimpedance were calculated relative to baseline. The data was examined in the whole image and in three (ventral, middle, dorsal) regions-of-interest per lung.

**Results:**

Compared with the baseline phase, chest electrical bioimpedance fell after the disconnection from the ventilator in all animals in both postures during all procedures. The fluid instillation further decreased electrical bioimpedance. During fluid recovery, electrical bioimpedance increased, but not to baseline values. All effects were highly significant (*p* < 0.001). The fractional changes in individual regions-of-interest were posture-dependent. The regional fall in electrical bioimpedance was smaller in the ventral and larger in the dorsal regions after the fluid instillation than after the initial disconnection to ambient pressure in supine animals (*p* < 0.001) whereas these changes were of comparable amplitude in prone position.

**Conclusions:**

The results of this study show a regionally dissimilar initial fall in electrical bioimpedance caused by non-uniform aeration loss at the beginning of the BAL procedure. They also confirm a further pronounced fall in bioimpedance during fluid instillation, incomplete recovery after suction and a posture-dependent distribution pattern of these effects.

**Electronic supplementary material:**

The online version of this article (10.1186/s40635-019-0225-6) contains supplementary material, which is available to authorized users.

## Background

The availability of electrical impedance tomography (EIT) devices certified for clinical use has increased the use of this method for functional chest imaging to continuously assess regional lung ventilation and changes in regional aeration in patients [1–4]. EIT is mainly utilised in mechanically ventilated patients in whom it provides an immediate feedback on the adequacy of chosen ventilator settings and early identification of adverse events like pneumothorax [5], pendelluft [6], alveolar overdistension and atelectasis [7–9] or cyclic recruitment [10].

EIT assesses the electrical properties of lung tissue and their regional variation depending on physiological and/or pathological changes in air volume. EIT probes the chest by rotating application of very small alternating electrical currents and measures the resulting electrical voltages. A change in local air volume, for instance, its increase during inspiration or alveolar recruitment, is associated with an elongation of pathways the current needs to pass through and this results in higher values of measured electrical bioimpedance. However, local fluid content also makes a contribution to regional pulmonary electrical bioimpedance. For instance, accumulation of fluid in the lung tissue, as in lung oedema [11] or in the pleural space, as in empyema [12] or pleural effusion [13], can be detected by EIT as a reduction in bioimpedance.

A recent report has tested the ability of EIT to determine regional changes in electrical bioimpedance resulting from instillation of fluid into the airways of patients undergoing diagnostic bronchoscopic and blind bronchoalveolar lavage (BAL) procedure [14]. The authors reported increases in electrical bioimpedance in all studied patients during BAL. Positive deflections in regional EIT waveforms and regions of high positive impedance change in functional EIT images were claimed to coincide with the moment of lavage fluid instillation. These findings are surprising because instillation of an electrically conductive saline into the lungs would have rather been expected to lower electrical bioimpedance than increase it.

Given that to our knowledge no other studies on the instantaneous effects of BAL on EIT findings currently exist, we have analysed a large data set of EIT recordings acquired in experimental animals during a total of 113 standardised BAL procedures. The experiments were conducted in a controlled environment without any interference that may occur in the ICU setting. We thus expected that our results would provide definitive information about the impact of BAL on bioimpedance within the lung and clarify what alterations might be expected on EIT recordings during BAL in the clinical setting.

## Methods

The study was approved by the Committee for Animal Care at the University of Geneva, Switzerland (protocol number 03-63, approval number 31.1.1051/2230/I) and adhered with the guidelines on animal experimentation. The experiments were designed to examine the effects of surfactant administration and alveolar recruitment on regional lung ventilation, aeration and respiratory system mechanics in an animal model of infant respiratory distress syndrome. These results have been reported before [15, 16]. Repeated BAL procedures were performed to induce acute lung injury, and EIT data was obtained during each BAL in 13 animals. This data has not been analysed before and is reported here for the first time.

### Animal preparation

Detailed description of animal preparation and instrumentation is provided in [15, 16]. Briefly, the animals were premedicated with midazolam and atropine. General anaesthesia was induced by ketamine and maintained with midazolam and fentanyl. Muscle paralysis was achieved by continuous infusion of pancuronium. The animals were tracheotomised, intubated and mechanically ventilated (Galileo Gold; Hamilton Medical, Switzerland). Arterial, venous and urinary catheters were placed. The pressure at the airway opening and airflow were monitored continuously (Florian respiratory monitor; Acutronic Medical Systems, Zug, Switzerland) and arterial partial pressures of oxygen (P_a_O_2_) and carbon dioxide (P_a_CO_2_) recorded (Paratrend 7; Diametrics Medical, High Wycombe, UK). EIT examinations were carried out at a scan rate of 13 images/s using alternating currents of 5 mA_rms_ and 70 kHz (Goe-MF II EIT system; Viasys Healthcare, Höchberg, Germany).

### Experimental protocol

The intubated animals were ventilated in a pressure-controlled mode with a fraction of inspired O_2_ of 1.0 throughout. The initial settings before the first BAL are given in Additional file 1: Table S1, along with the values recorded half an hour after the last BAL.

All whole-lung BAL procedures were performed in a standardised way using warmed saline (50 ml/kg body weight). The criterion for termination of the procedures was stable P_a_O_2_ lower than 100 mmHg for 30 min. The first four lavages in each animal were performed in the supine posture, the next series of up to four in the prone position, with any further lavages in supine.

**Fig. 1:**
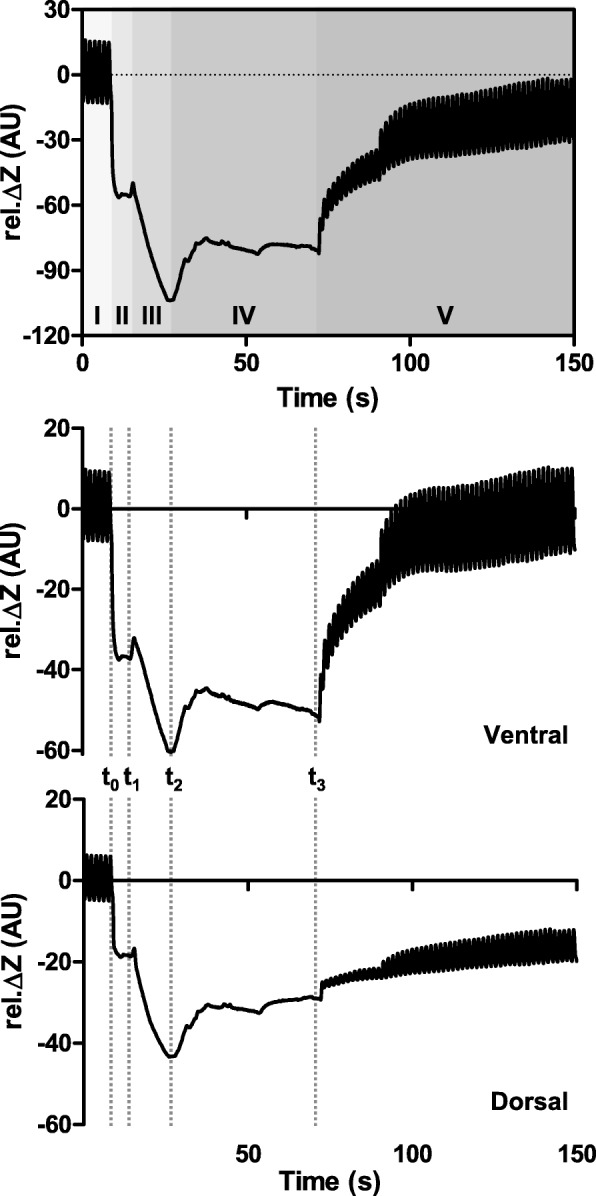
Global and regional EIT waveforms with highlighted measurement phases before, during and after bronchoalveolar lavage. The global waveform shows the sum of all image pixel values of relative impedance changes (rel. Δ*Z*) in arbitrary units (AU) (top), the regional ones in the ventral (middle) and dorsal image sections (bottom). The recording continued through five phases: I, continuous mechanical ventilation; II, disconnection of the endotracheal tube from the ventilator; III, administration of the lavage fluid through the endotracheal tube; IV, fluid recovery by suction, V, re-connection of the endotracheal tube to the ventilator and resumption of mechanical ventilation. (The small dent in the waveforms after approximately 60% of the suction phase IV (i.e. at 52 s of the recording) was associated with the temporary evacuation of the fluid from the suction syringe. The clearly discernible change in the waveforms with higher tidal variation amplitude and increased end-expiratory values after the resumption of ventilation in phase V at time point 89 s resulted from the adjustment in ventilator settings with higher PEEP.) EIT data was analysed at three standardised time points: *t*_1_, after disconnection from the ventilator directly prior to the fluid instillation into the lungs; *t*_2_, at the end of fluid instillation and *t*_3_, at the end of suction of the lavage fluid immediately before the resumption of mechanical ventilation, in each case compared with bioimpedance at baseline phase I prior to the disconnection from the ventilator at *t*_0_

EIT data was recorded for 150 s during each BAL procedure. The recording started shortly before the disconnection of the endotracheal tube from the ventilator (*t*_0_) and covered the whole further period of fluid instillation, recovery of the fluid by suction and the initial phase of resumed mechanical ventilation (Fig. 1). After each BAL, positive end-expiratory pressure (PEEP) was transiently increased if necessary to assist in recovery from hypoxaemia.

To characterise the EIT findings during the BAL procedure three distinct reproducible time points were chosen: (1) after ventilator circuit disconnection, immediately before lavage fluid instillation (*t*_1_), (2) at the end of fluid instillation (*t*_2_) and at the end of fluid recovery by suction (*t*_3_) (Fig. 1.). EIT data was analysed in all 912 image pixels in the whole image, and in functional regions-of-interest [17] corresponding to the ventral, middle and dorsal areas of the left and right lung regions. The individual regions-of-interests were delineated using equidistant ventral to dorsal gridlines [15]. For each image pixel, change in EIT impedance was calculated relative to the baseline phase during mechanical ventilation (rel. Δ*Z*, dimensionless arbitrary units (AU)) at the three time points described above. The values of rel. Δ*Z* were then summed for all image pixels and within all six regions-of-interest.

### Statistical analysis

All statistical analyses were performed with GraphPad Prism 5.01 (GraphPad Software Inc., San Diego, CA, USA). Data is presented as mean ± SD, unless otherwise indicated. The data was first tested using the D’Agostino-Pearson normality test. To test the effect of the time point on electrical bioimpedance, repeated measures ANOVA with Bonferroni’s multiple comparison test or the Friedman test with subsequent Dunn’s multiple comparison were applied. To test the effect of body posture, one-way analysis of variance with Bonferroni’s multiple comparison test or the Kruskal-Wallis test with Dunn’s multiple comparison test were used. *p* values < 0.05 were considered significant.

## Results

The studied newborn piglets had a body weight of 2.2 ± 0.3 kg. In total, 113 BAL procedures were performed. Fifty-one BALs were accomplished in the supine, 46 in the prone and 16 in the repeated supine position. The individual numbers of lavages differed among the animals, the mean number per animal was 8.7 ± 1.8. (The general effects of the repeated BAL procedures on the respiratory system mechanics and pulmonary gas exchange are summarised in Additional file 1: Table S1.)

Figure 1 demonstrates the changes in electrical bioimpedance during the whole EIT recording during one BAL procedure: the ventilation-related breath-by-breath signal variation observed during mechanical ventilation in phase I, the rapid fall in electrical bioimpedance after the disconnection to ambient pressure (phase II), the slightly more protracted further fall during the lavage fluid instillation (phase III), the initial impedance increase during early suction that was slowed down and regionally even slightly reversed during later suction (phase IV) followed by the increase in impedance after the resumption of mechanical ventilation with reappearance of ventilation-related tidal impedance variation (phase V).

**Fig. 2:**
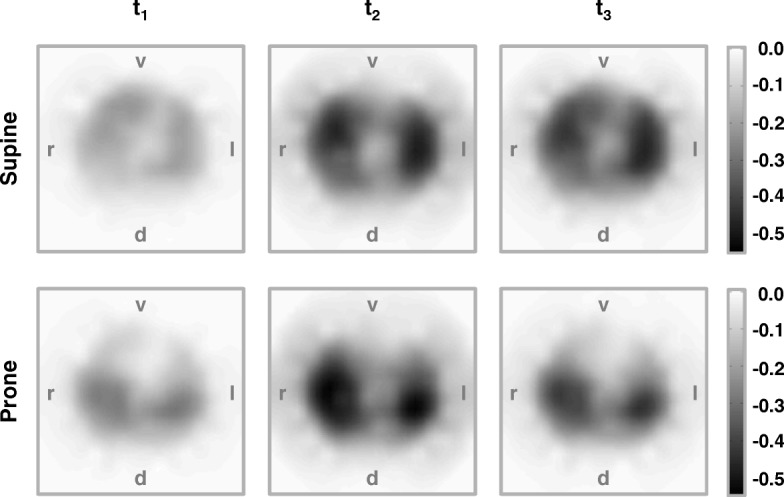
Example functional EIT images. The images show the regional fall in electrical bioimpedance in lung areas in one of the examined animals in the supine (top) and the prone positions (bottom) at three time points during the bronchoalveolar lavage procedure. The impedance decrease is shown in dark tones after the disconnection of the endotracheal tube from the ventilator before the fluid instillation (*t*_1_), after the fluid instillation (*t*_2_) and after the fluid suction before the resumption of ventilation (*t*_3_) in each case relative to baseline. v, ventral; d, dorsal; r, right; l, left

The typical changes in regional electrical bioimpedance during the BAL procedure in the supine and prone positions at the three time points are readily discernible in exemplary functional EIT images (Fig. 2). After the disconnection from the ventilator, a fall in lung impedance was noted in the lung regions, especially in the non-dependent areas (i.e. the ventral ones in the supine and dorsal ones in the prone position). The instillation of the lavage fluid led to a further pronounced decrease in electrical bioimpedance in all lung regions. The recovery of fluid by suction slightly increased electrical bioimpedance, but not to pre-lavage values.

The analysis of all 113 EIT data sets revealed that the three distinct time points during the BAL procedure at which EIT data were quantitatively evaluated were reproducible among the animals. The time point *t*_1_ occurred at 4.8 (4.0–6.2) s (median (interquartile range)) after disconnection from the ventilator, *t*_2_ was 21.0 (18.3–23.8) s beyond *t*_0_, and *t*_3_ at 70.8 (60.0–79.2) s.

**Fig. 3:**
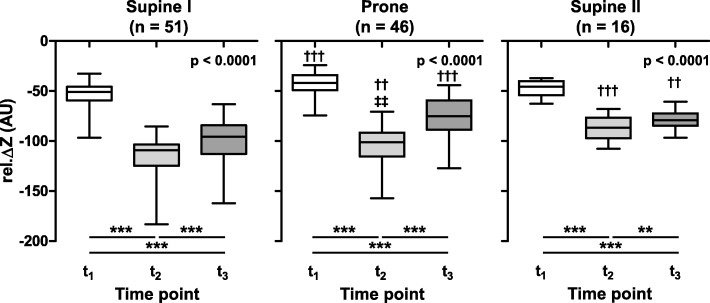
Change in pulmonary electrical bioimpedance at distinct time points during the bronchoalveolar lavage procedure. The sums of all image pixel values of relative impedance changes (rel. Δ*Z*) in arbitrary units (AU) are given after the disconnection of the endotracheal tube from the ventilator before the lavage fluid instillation (*t*_1_), after the fluid instillation (*t*_2_) and after the fluid suction before the resumption of ventilation (*t*_3_), in each case compared to baseline before the disconnection to ambient pressure. The data originate from lavages performed first in supine (left), then prone (middle) and final supine (right) positions (supine I, prone and supine II, respectively). The numbers of analysed bronchoalveolar lavages in each posture are given at the top of each diagram. *p* values given in the diagrams highlight the highly significant effect of the time point. The significance of differences between the individual time points was obtained from post analyses at **p < 0.01 and ***p < 0.001. Significantly different values from the supine I position at corresponding time points are given as ††*p* < 0.01 and †††*p* < 0.001 and from supine II position as ‡‡*p* < 0.01

The sums of all calculated pixel values of rel. Δ*Z* obtained for all data sets in the supine I, prone and supine II positions at *t*_1_, *t*_2_ and *t*_3_ show a highly consistent pattern: a decrease in bioimpedance (fall in rel. Δ*Z*) after disconnection from the ventilator, a further significant fall after the fluid instillation, and an increase after suction (Fig. 3). The rel. Δ*Z* values remained significantly lower by the end of fluid recovery than after the initial disconnection to ambient pressure. Overall, positive impedance changes compared with the baseline phase of initial mechanical ventilation were not observed in any of the BAL procedures in either animal at any of the three time points.

**Fig. 4:**
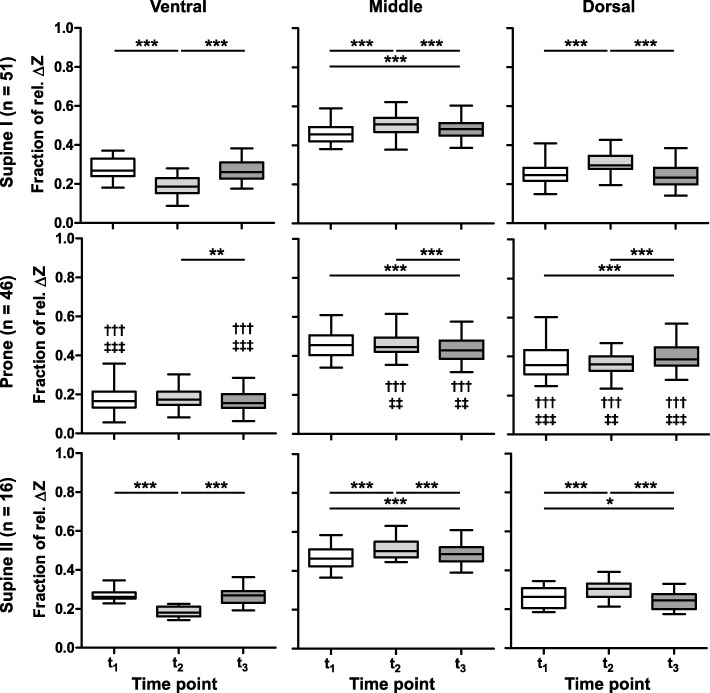
Fractional bioimpedance change in ventral, middle and dorsal regions-of-interest. The fractional changes are given at three time points during the bronchoalveolar lavage procedure: after the disconnection of the endotracheal tube from the ventilator before the lavage fluid instillation (*t*_1_), after the fluid instillation (*t*_2_) and after the fluid suction before the resumption of ventilation (*t*_3_). The data was obtained first in supine (top row), then prone (middle row) and supine (bottom row) animals (supine I, prone and supine II, respectively). The significance of differences between the individual time points was derived from post analyses at *p < 0.05, **p < 0.01 and ***p < 0.001. Significantly different values from the supine I position at corresponding time points are given as †††*p* < 0.001 and from supine II position as ‡‡*p* < 0.01 and ‡‡‡*p* < 0.001

The regional changes in rel. Δ*Z* in the ventral, middle and dorsal regions-of-interest at *t*_1_, *t*_2_ and *t*_3_, given as fractions of the overall impedance changes in the lung regions, exhibited different distribution patterns (Fig. 4). The fractional changes in rel. Δ*Z* were larger in the ventral regions in the supine posture after the disconnection to ambient pressure at *t*_1_ and fluid recovery at *t*_3_ than after fluid instillation at *t*_2_ (Fig. 4, left top and left bottom panels). In the dorsal regions, the fractional change in rel. Δ*Z* was the largest at *t*_2_ (Fig. 4, right top and right bottom panels). These effects were partially reversed in the prone posture but less marked (Fig. 4, middle row of panels). The fractional impedance changes were significantly lower in the ventral regions in the prone than in both supine positions at time points *t*_1_ and *t*_3_ (Fig. 4, left middle panel) and significantly higher in the dorsal regions (Fig. 4, right middle panel). The regional evaluation also confirmed that only negative values of rel. Δ*Z* (i.e. decreasing bioimpedance) were found in all regions-of-interest during all BAL procedures at all three studied time points compared with the baseline phase before *t*_0_.

## Discussion

The results of our study showed that the instillation of fluid into the lungs during BAL led to a consistent pronounced decrease in electrical bioimpedance that could be visualised and quantitatively assessed by EIT. It occurred in the whole left and right lung regions with spatially different distribution patterns in the supine and prone positions. This data is novel since the instantaneous effects of BAL on chest EIT findings have not been described before in spite of its previous use to induce experimental acute lung injury (e.g. [18–21]).

Since the lavage fluid was instilled in an open procedure and EIT data acquisition began before the disconnection from the ventilator, we could examine the effects of exposure to ambient pressure on regional electrical bioimpedance. Each disconnection led to a fall in rel. Δ*Z* resulting from a loss in gas volume. This corresponds to earlier EIT findings obtained in supine pigs [18]. The regional effects were posture-dependent, showing a higher fraction of rel. Δ*Z* fall in the ventral regions in the supine than in prone animals with an opposite behaviour in the dorsal regions. Our data also showed a smaller decrease in bioimpedance in the prone than in the supine position indicating a less pronounced relative aeration loss in this posture.

The recovery of the fluid by suction led to an increase in electrical bioimpedance, but not to the values seen prior to the fluid instillation. This can be attributed not only to the incomplete recovery of the lavage fluid but also to de-recruitment and further aeration loss caused by suction. These effects differed regionally and exhibited a posture-dependent behaviour. Although a few studies exist reporting the use of EIT to analyse the effects of endotracheal tube suctioning on lung aeration [18, 22–25], they cannot be directly compared with our data, which reports specifically on the effects noted during large volume saline lavage. Nevertheless, these previous studies reported a fall in global rel. Δ*Z* during suctioning compared with the preceding baseline phase of mechanical ventilation [23, 24]. The steepest fall in rel. Δ*Z* was found in dorsal lung regions in supine animals [18]. This was attributed to larger suction-induced de-recruitment in the dependent regions. Tingay et al. [25] examined the effects of open and closed suction in ventral and dorsal regions in supine piglets showing that pronounced regional fall in rel. Δ*Z* during suction could only be prevented by closed suction using a small-calibre catheter. In our study, suctioning removed the free fluid from the lungs, and this led to an increase in rel. Δ*Z* mainly in the dependent lung where the fluid had accumulated. By the end of the fluid recovery, the non-dependent regions experienced a fall in rel. Δ*Z* in supine animals, highlighting the deleterious effects of suction on lung aeration.

A recent clinical study, which prompted our systematic analysis of EIT findings obtained during BAL procedures, evaluated bioimpedance in patients undergoing bronchoscopic and blind BALs [14]. In that study, transient increases in electrical bioimpedance were identified in the EIT waveforms and attributed to the instillation of lavage fluid. Regions of high positive rel. Δ*Z* change, identified in the functional EIT images of the studied patients during the BAL procedures, were considered to represent the lung regions with fluid accumulation during the instillation. Our findings are clearly contradictory to these clinical observations, with instillation of lavage fluid consistently producing a decrease in rel. Δ*Z*. Our results are in harmony with theoretical considerations in relation to electrical resistivity of body tissues and sodium chloride solutions. The electrical resistivity of lung tissue in vivo is generally given at about 750 to 2100 Ω cm (depending on the frequency of excitation currents, temperature, species and degree of lung inflation) [26, 27] whereas warm 0.9% saline at body temperature has a resistivity of 51 Ω cm [26]. The instillation of a highly electrically conductive crystalloid solution into the less conductive lung must therefore lead to a decrease in the chest electrical bioimpedance measured by EIT, and our results unambiguously confirm this.

Why a theoretically anticipated fall in electrical bioimpedance was not identified in the previous study in patients during BAL [14] could be explained as follows. Based on the original screen-captured EIT waveforms and the described methodological approach with the signals ‘later reviewed by three different physicians with no expertise in EIT who were blind to the procedures performed’, we presume that the blinded reviewers performing the offline analyses of the data erroneously identified the largest positive peaks in electrical bioimpedance signals as being associated with BAL. They selected these as representing the effects of fluid instillation and used them to generate functional EIT images of the presumed effects of fluid instillation on the spatial distribution of electrical bioimpedance in the chest cross-section.

These infrequent positive peaks could have theoretically resulted from spontaneous breaths; however, the patients were described as sedated and paralysed for the procedure. They also could have been caused by undocumented manual inflations. In that case, the functional images generated from these peaks would rather show the inflation of lung regions unobstructed by the catheter or bronchoscope than the lavaged region.

Finally, we would like to address a few limitations of our study: (1) EIT scanning was performed at a relatively low scan rate compared with the most modern EIT devices. However, the rate of 13 images/s was more than sufficient to reliably identify the relevant time points and obtain the corresponding data needed for the analysis. (2) We have performed only open BAL procedures. It can be presumed that a closed procedure would not result in a pronounced fall in electrical bioimpedance seen in our data after the disconnection to ambient pressure as previously shown [25]. It is probable that the rel. Δ*Z* values after the closed suctioning might have also been different as can be implied from a previous study [24]. We expect that the effects of fluid instillation should be similar in open and closed BAL; however, the present data do not allow to confirm this.

## Conclusions

Our study has described for the first time the global and regional pulmonary effects of lavage fluid instillation and subsequent suctioning in an in vivo animal model using EIT. A marked fall in chest electrical bioimpedance resulted from BAL which was only partially reversed by fluid recovery by suction. The spatial distribution of these effects was posture-dependent. Based on our results, we recommend re-evaluation of the effects of BAL on bioimpedance in the clinical setting.

## Additional file

Additional file 1: **Table S1.** Ventilator settings, respiratory mechanics and blood gases. (DOCX 31 kb)

## Abbreviations

AU: Arbitrary units
BAL: Bronchoalveolar lavage
EIT: Electrical impedance tomography
*f*_resp_: Respiratory rate
ICU: Intensive care unit
P_a_CO_2_: Arterial partial pressure of carbon dioxide
P_a_O_2_: Arterial partial pressure of oxygen
PEEP: Positive end-expiratory pressure
PIP: Peak inspiratory pressure
rel. Δ*Z*: Relative impedance change
SD: Standard deviation
*t*_0_: Time point of disconnection from the ventilator
*t*_1_: EIT data analysis time point after disconnection from the ventilator directly prior to the fluid instillation into the lungs
*t*_2_: EIT data analysis time point at the end of lavage fluid instillation
*t*_3_: EIT data analysis time point at the end of suction of the lavage fluid immediately before the resumption of mechanical ventilation
*V*_T_: Tidal volume
