# Supplementary material for: Regional pulmonary effects of bronchoalveolar lavage procedure determined by electrical impedance tomography
Source: Intensive Care Med Exp. 2019 Feb 15;7:11. doi: 10.1186/s40635-019-0225-6 (PMC6377686; doi:10.1186/s40635-019-0225-6)
Supplement: Supplementary file 1 — Table S1. Ventilator settings, respiratory mechanics and blood gases. (DOCX 31 kb) [file 40635_2019_225_MOESM1_ESM.docx]

**Additional file 1**

**Table S1** Ventilator settings, respiratory mechanics and blood gases

|  | Before BAL | After BAL |
| --- | --- | --- |
| V_T_ (ml/kg BW) | 11.3 ± 1.0 | 9.9 ± 1.3* |
| PEEP (cmH_2_O) | 5.0 ± 0.3 | 4.9 ± 1.0 |
| PIP (cmH_2_O) | 13.0 ± 1.4 | 20.9 ± 4.6*** |
| f_resp_ (/min) | 34.6 ± 6.0 | 46.1 ± 8.0*** |
| C_rs_ (ml/cmH_2_O/kg BW) | 1.6 ± 0.3 | 0.7 ± 0.2*** |
| R_rs_ (cmH_2_O/ml/s/kg BW) | 0.027 ± 0.004 | 0.039 ± 0.006*** |
| P_a_O_2_ (mmHg) | 442 ± 46 | 63 ± 18*** |
| P_a_CO_2_ (mmHg) | 48 ± 6 | 52 ± 9 |

Data are reported as mean ± standard deviation

*BAL* bronchoalveolar lavage; *V_T_* tidal volume; *BW* body weight; *PEEP* positive end-expiratory pressure; *PIP* peak inspiratory pressure; *f_resp_* respiratory rate; *C_rs_* respiratory system compliance; *R_rs_* respiratory system resistance; *P_a_O_2_* arterial partial pressure of oxygen; *P_a_CO_2_* arterial partial pressure of carbon dioxide. Significantly different values from before BAL are given as * p<0.05; *** p<0.001. (The significance of differences between the ventilator settings, respiratory system mechanics and blood gases before and after the sequence of BAL procedures was tested with the paired t test or the Wilcoxon signed rank test as appropriate.)
